# Supplementary material for: Evidence supported by Mendelian randomization: impact on inflammatory factors in knee osteoarthritis
Source: Front Med (Lausanne). 2024 May 28;11:1382836. doi: 10.3389/fmed.2024.1382836 (PMC11165061; doi:10.3389/fmed.2024.1382836)
Supplement: Supplementary file 1 [file Data_Sheet_1.ZIP › data/GCST90274811_5e6/811.leaveoneout.pdf]

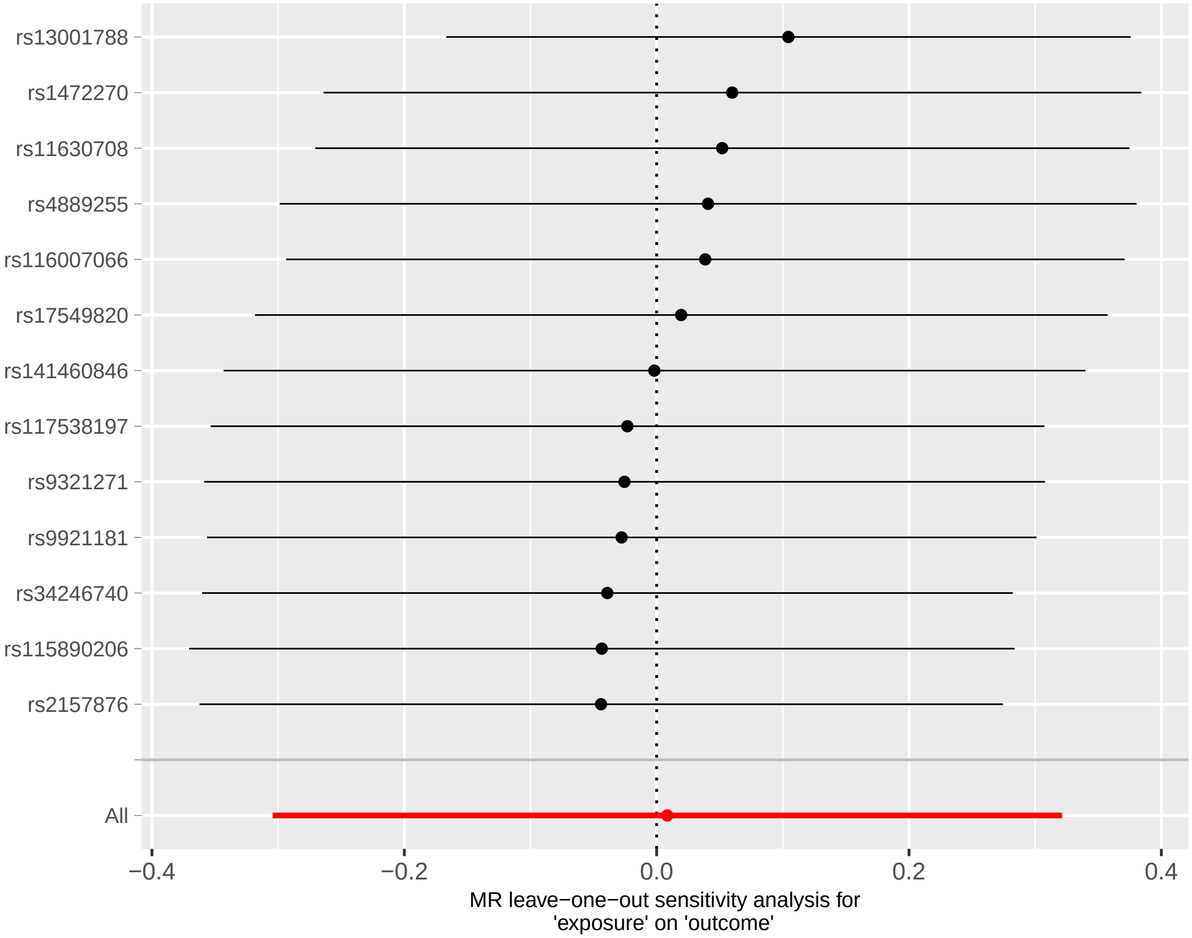
rs13001788
rs1472270
rs11630708
rs4889255
rs116007066
rs17549820
rs141460846
rs117538197
rs9321271
rs9921181
rs34246740
rs115890206
rs2157876
All
−0.4
−0.2
0.0
0.2
0.4
MR leave−one−out sensitivity analysis for
'exposure' on 'outcome'
